# Hypervitaminosis A is prevalent in children with CKD and contributes to hypercalcemia

**DOI:** 10.1007/s00467-014-2916-2

**Published:** 2014-08-15

**Authors:** Baheerathi Manickavasagar, Andrew J. McArdle, Pallavi Yadav, Vanessa Shaw, Marjorie Dixon, Rune Blomhoff, Graeme O’ Connor, Lesley Rees, Sarah Ledermann, William van’t Hoff, Rukshana Shroff

**Affiliations:** 1 Department of Dietetics, Great Ormond Street Hospital for Children NHS Foundation Trust, London, United Kingdom; 2 Nephrology, Great Ormond Street Hospital for Children NHS Foundation Trust, London, United Kingdom; 3 Department of Nutrition, University of Oslo, Oslo, Norway

**Keywords:** Vitamin A, Retinol, Retinol binding protein 4 (RBP4), Hypercalcemia, Children

## Abstract

**Background:**

Vitamin A accumulates in renal failure, but the prevalence of hypervitaminosis A in children with predialysis chronic kidney disease (CKD) is not known. Hypervitaminosis A has been associated with hypercalcemia. In this study we compared dietary vitamin A intake with serum retinoid levels and their associations with hypercalcemia.

**Methods:**

We studied the relationship between vitamin A intake, serum retinoid levels, and serum calcium in 105 children with CKD stages 2–5 on dialysis and posttransplant. Serum retinoid measures included retinol (ROH), its active retinoic acid (RA) metabolites [all-trans RA (at-RA) and 13-cis RA] and carrier proteins [retinol-binding protein-4 (RBP4) and transthyretin (TTR)]. Dietary vitamin A intake was assessed using a food diary.

**Results:**

Twenty-five children were in CKD 2–3, 35 in CKD 4–5, 23 on dialysis and 22 posttransplant; 53 % had vitamin A intake above the Reference Nutrient Intake (RNI) value. Children receiving supplemental feeds compared with diet alone had higher vitamin A intake (*p* = 0.02) and higher serum ROH (*p* < 0.001). Notably, increased ROH was seen as early as CKD stage 2. For every 10 ml/min/1.73 m^2^ fall in estimated glomerular filtration rate (eGFR), there was a 13 % increase in ROH. RBP4 levels were increased in CKD 3–5 and dialysis patients. The lowest ratios of ROH:RBP4 were seen in dialysis compared with CKD 2–3 (*p* = 0.03), suggesting a relative increase in circulating RBP4. Serum ROH, RBP4 and at-RA were associated with serum calcium. On multivariable analysis RBP4 levels and alfacalcidol dose were significant predictors of serum calcium (model *R* ^2^ 32 %) in dialysis patients.

**Conclusions:**

Hypervitaminosis A is seen in early CKD, with highest levels in children on supplemental feeds compared with diet alone. Serum retinoid levels significantly predict hypercalcemia.

## Introduction

**Fig. 1:**
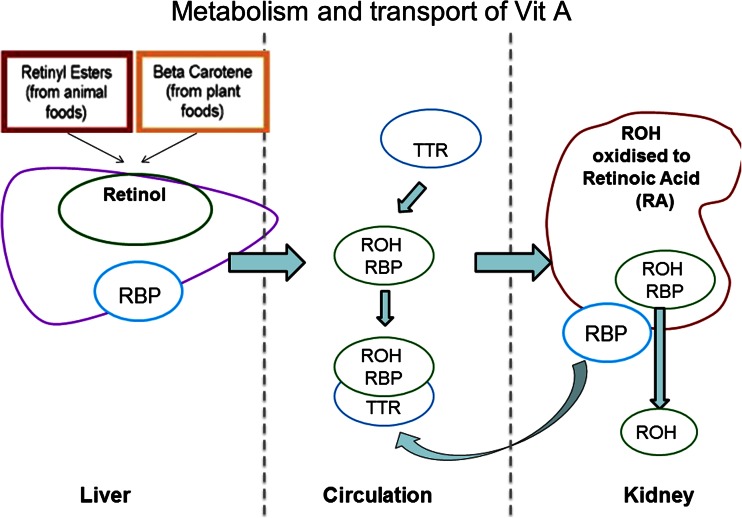
Metabolism, transport, and degradation of vitamin A under physiological conditions. *RBP* retinol binding protein, *ROH* retinol, *TTR* transthyretin

The kidneys play an important role in the metabolism and excretion of vitamin A [1, 2]. In healthy individuals, dietary vitamin A is converted into retinol (ROH), stored in the liver and transported by its carrier proteins—retinol-binding protein-4 (RBP4) and transthyretin (TTR)—to its target cells [1]. ROH is oxidized to its active form, retinoic acid (RA), and free RBP4 (apo-RBP) is then degraded and filtered by the kidneys [2]. Patients with impaired renal function have been shown to have high circulating levels of ROH, possibly due to a combination of decreased glomerular filtration of the ROH-RBP4 complex, reduced conversion of ROH to RA [2], and an accumulation of RBP4 [1, 3, 4] (Fig. 1). Studies in adults [5–7] and children [8–10] on dialysis have shown increased ROH and RBP4 levels. However, little is known about vitamin A status at earlier stages of chronic kidney disease (CKD), its association with dietary intake, or the consequences of high circulating vitamin A metabolites.

In the absence of evidence-based guidelines on vitamin A intake in patients with CKD, accepted practice in the UK is to avoid exceeding twice the Reference Nutrient Intake (RNI) value [11]. The Kidney Disease Outcomes Quality Initiative (KDOQI) guideline recommends that the total dietary vitamin A intake should be limited to the Dietary Reference Intake (DRI) value [12], the US equivalent to RNI value [13]. These guidelines are opinion based, with no studies in the pediatric CKD population to support them. This is difficult to achieve in practice, since commercial nutritional supplements used in CKD patients are fortified with vitamin A.

In our clinical practice, we have observed hypercalcemia in some children with CKD even without the use of calcium or vitamin D supplements, and hypothesized that vitamin A in supplemental feeds was associated with hypercalcemia. Indeed, case reports have suggested that high ROH levels are associated with hypercalcemia, both in individuals with normal renal function [14, 15] and dialysis patients [16–18]. Also, retinoid levels have been linked to an increased risk of subclinical cardiovascular disease in the general population [19–21]. In animal models, pharmacological doses of vitamin A suppress osteoblast activity [22] and increase osteoclast formation and differentiation [23], which together manifest as osteopenia, fractures, bony deformities, and growth arrest and lead to a release of calcium from the bones. Hypercalcemia is a well-established risk factor for vascular calcification, which, in turn, contributes to a high cardiovascular mortality in adults and children with CKD [24–26]. In this study, we compared dietary vitamin A intake with serum retinoid levels and their associations with hypercalcemia.

## Materials and methods

We recruited 105 consecutive children under 18 years of age with CKD stages 2–5 [estimated glomerular filtration rate (eGFR) < 90 ml/min/1.73 m^2^), on dialysis, or with a functioning renal transplant (≥ 3 months posttransplant) from nephrology outpatient clinics at Great Ormond Street Hospital NHS Foundation Trust (Table 1). Children with active liver disease or receiving fat-soluble multivitamin supplements that can influence vitamin A status were excluded. Informed written consent from parents or caregivers, and assent from children where appropriate, was obtained. The study was approved by a local research ethics committee.

**Table 1:** Patient demographics and biochemical data

	CKD 2–3 (*n* = 25)	CKD 4–5 (*n* = 35)	Dialysis (*n* = 23)	Transplant (*n* = 22)	*P* value
Age (years)	3.3 (1.6–7.8)	9.0 (3.0–14.1)	8.6 (3.1–12.0)	13.7 (8.6–16.9)	<0.001
Male [*n* (%)]	16 (64 %)	21 (60 %)	15 (65 %)	11 (50 %)	
Race (*n*) Arabic/Asian/Afro-Caribbean/Caucasian/mixed	1/7/3/13/1	1/7/4/21/2	1/5/2/14/1	1/7/0/13/1	
Underlying diagnosis (*n*) congenital anomalies of the kidneys and urinary tract (CAKUT), glomerular diseases, congenital nephrotic syndrome, polycystic kidney disease	22/1/0/2	34/1/0/0	9/9/5/0	18/2/11	
Body mass index (BMI) SDS	0.9 (−0.2 to 1.5).	0.7 (−1.1–1.4)	−0.2 (−1.8 to 0.8)	1.3 (0.2–1.7)	0.06
eGFR* (ml/min/1.73 m^2^)	52.5 (35.0–65.1)	15.4 (12.9–21.5)	–	63.7 (39.0–87.4)	
Dialysis type PD or HD [*n* (%)]			11 (48 %) 12 (52 %)		
Time on dialysis (months)			8.2 ± 7.5		
Time posttransplant (years)				2.7 (1.3–5.3)	
Biochemical data					
Calcium (albumin-adjusted)** (mmol/L)	2.42 (2.33–2.49)	2.43 (2.36–2.49)	2.52 (2.43–2.69)	2.36 (2.32–2.42)	<0.001
Phosphate (mmol/L)	1.52 (1.39–1.66)	1.58 (1.33–1.73)	1.57 (1.3–1.92)	1.16 (1.08–1.43)	0.001
Parathyroid hormone (pmol/L)	5.3 (3.3–6.8)	5.8 (2.5–11.1)	14.3 (1.4–38.4)	5.4 (3.5–7.0)	0.03
Alkaline phosphatase (U/L) (in 94 children)	257 (203–317)	284 (208–396)	241 (190–395)	172 (95–196)	<0.001
25 (OH) D (nmol/L)	48 (26–94)	31 (22–71)	26 (11–51)	66 (45–73)	0.34
Medications					
Use of phosphate binder, calcium-based binder [*n* (%)], non-calcium-based binder [*n* (%)], total calcium intake (diet, feed, and binders; mg/day)	10 (40 %), 0 (0 %), 753 (560–1,268)	24 (69 %), 1 (3 %), 1,018 (659–1,352)	19 (83 %), 5 (22 %), 1,768 (383–2,232)	0 (0 %), 0 (0 %), 852 (516–984)	<0.001, 0.004, 0.07
Vitamin D prescription, use of alfa-calcidiol [*n* (%)], use of cholecalciferol; *n* (%)], alfacalcidol dosage (μg) (in 72/105 children)	14 (56 %), 3 (12 %), 0.4 (0–0.5)	24 (69 %), 1 (3 %), 0.38 (0.1–1.0)	17 (74 %), 0 (0 %), 0.53 (0.1–1.4)	11 (50 %), 3 (14 %), 0.2 (0–0.42)	0.29, 0.14, 0.15

Data are expressed as median (interquartile range). *P* values are for comparison across all groups obtained from one-way analysis of variance (ANOVA) or Kruskal–Wallis test, as appropriate

*SDS* standard deviation score, eGFR estimated glomerular filtration rate, *CKD* chronic kidney disease, *PD* peritoneal dialysis, *HD* hemodialysis

*Calculated using the modified Schwartz formula [46]

**Albumin-adjusted calcium was calculated by the formula [(40 − observed albumin) x 0.025] + observed calcium

All clinical, anthropometric, and biochemical data were collected at the time of a single outpatient visit. Doses of phosphate binders and vitamin D supplements were noted from clinic records, and calcium intake from phosphate binders was calculated.

### Biochemical analysis

Routine serum biochemistry including creatinine, calcium, phosphate, parathyroid hormone (PTH) (Immulite 2500 Intact PTH assay; Siemens), and 25-hydroxyvitamin D [(25(OH)D, measured by isotope-dilution liquid chromatography–tandem mass spectrometry (LC-MS)] were measured. Blood samples for vitamin A metabolites ROH, carrier proteins RBP4, TTR, and RA [all-trans RA (at-RA) and 13-cis RA] were frozen at −80 °C and analyzed in batches. Serum ROH was measured by high-performance liquid chromatography (HPLC) with UV detection in the Chemical Pathology Laboratory at Great Ormond Street Hospital. RBP4 and TTR were measured by nephelometry (Siemens BN2 nephelometer) at the Sheffield Protein Reference Unit, UK. Serum 13-cis-RA and at-RA were measured in 53 randomly selected children (CKD 2–3 *n* = 13, CKD 4–5 *n* = 20, dialysis *n* = 5, transplant *n* = 15) by LC-MS at Vitas, Oslo Innovation Centre, Norway [27]. The molar ratios of ROH:RBP4 and RBP4:TTR were calculated. Patients were compared with 12 healthy age- and gender-matched children attending ear nose and throat (ENT) clinics at our hospital. Normal levels of ROH are well established and are age specific, as outlined in Table 2. RBP4 and TTR levels were measured (Table 2). Only adult reference ranges for at-RA were available at the time of the study and are 0.9–2.0 ng/ml.

**Table 2:** Dietary vitamin A intake and serum levels

	Normal reference range	CKD 2–3 (*n* = 25)	CKD 4–5 (*n* = 35)	Dialysis (*n* = 23)	Transplant (*n* = 22)	*P* value
Vitamin A intake data available [*n* (%)[		19 (76 %)	25 (71 %)	21 (91 %)	7 (32 %)	
Vitamin A intake (µg/day)		486 (422–795)	418 (329–509)	440 (315–579)	459 (382–649)	0.45
Serum ROH (µmol/l)	1–6 years 0.7–1.5; 6–12 years 0.9–1.7; 12–19 years 0.9–2.5	2.26 (1.72–2.57)	3.21 (2.37–3.77)	3.79 (3.09–5.32)	1.99 (1.65–2.48)	<0.001
13 cis-RA (ng/ml) (*n* = 53)	0.9–2.0^a^	0.46 (0.36–0.61)	0.47 (0.34–0.52)	0.45 (0.4–0.45)	0.41 (0.32–0.49)	0.41
at-RA (ng/ml) (*n* = 53)	0.9–2.0^a^	0.46 (0.4–0.58)	0.56 (0.46–0.68)	0.65 (0.64–0.74)	0.48 (0.4–0.49)	<0.001
Serum RBP4 (µmol/l)	0.94–2.41	2.83 (2.17–3.57)	4.67 (4.00–5.40)	6.84 (6.42–8.61)	2.15 (1.76–2.98)	<0.001
Serum TTR (µmol/l)	1.93–5.22	3.38 (2.92–3.69)	4.62 (4.15–5.38)	4.92 (4.31–5.54)	3.46 (3.00–3.85)	<0.001
ROH:RBP4		0.81 (0.71–0.88)	0.72 (0.56–0.82)	0.59 (0.41–0.73)	0.92 (0.79–1.00)	0.03
RBP4:TTR		0.82 (0.82–0.94)	1.00 (0.89–1.13)	1.44 (1.35–1.72)	0.70 (0.56–0.83)	0.02

Data are expressed as median (interquartile range) *P* values are for comparison across all groups obtained from one-way analysis of variance (ANOVA) or Kruskal–Wallis test, as appropriate

*CKD* chronic kidney disease, *ROH* retinol, *13 cis-RA* 13-cis Retinoic acid, *at-RA* all-trans retinoic acid, *TTR* transthyretin

^a^Adult reference ranges for at-RA

### Dietary analysis

A food diary and details of supplementary feeds were requested for 3 nonconsecutive days. Completed food diaries were obtained for 72 (69 %) children. Children were divided into three groups based on the level of nutritional support they consistently received: feed only, diet and supplementary feed, or diet alone. Feed products used were standard infant, infant–pediatric, or adult-renal-specific feeds. Feeds were adjusted to control biochemistry and meet full nutritional requirements; it is not possible to titrate feeds based upon recommended vitamin A intakes alone. Feeds were given either orally or via a nasogastric or gastrostomy tube. Patients on diet alone were advised on protein, phosphate, and potassium intakes, as appropriate, for their CKD stage as per KDOQI guidelines [12].

The intake of food from food diaries was expressed in grams or milliliters (taken from original food labels where appropriate). Software packages COMPEAT (Nutrition Systems, Banbury, UK) and Electronic Dietetics Manager (edm2000, Milton Keynes, UK) were used for analyses of dietary vitamin A intake from diet and supplemental feeds. Dietary ROH equivalents included provitamin A carotenoids from plant sources and retinyl esters from animal sources and were collectively grouped and expressed as the equivalent mass of retinol. Dietary vitamin A intake was also expressed as a percentage of RNI (defined as the amount of a nutrient that is sufficient to meet the nutritional needs of 97.5 % of a population of healthy individuals) [13]. The RNI for vitamin A is age and gender specific, as follows: 350 μg (0–12 months), 400 μg (1–6 years), 500 μg (7–10 years), 600 μg (11–14 years), 600 μg (15–18 years in females), and 700 μg (15–18 years in males). The USA Institute of DRI is similar [28]: 300 µg (1–3 years), 400 μg (4–8 years), 600 μg (9–13 years), and 750 μg (14–18 years). Dietary vitamin D, calcium, and phosphate were also calculated from the food diaries. All dietary calculations were performed by a single experienced renal dietician who was blinded to patients’ CKD status.

### Statistical analysis

Data were analyzed using SPSS, version 19 (SPSS, Chicago, IL, USA). As most variables had non-normal distributions, data were expressed as medians and interquartile ranges (IQR). Tests for difference in medians between groups were performed by Wilcoxon rank-sum test. Simple linear regression was used to test for associations between serum concentrations of ROH and its metabolites and ROH with eGFR; logarithms of concentrations were used to capture the nature of the relationship. Simple linear regression was used to investigate associations between serum albumin-adjusted calcium concentration and candidate predictors. Variables with *p* values < 0.15 were included in a stepwise multiple linear regression analysis. *P* < 0.05 was taken to be statistically significant.

## Results

Clinical and biochemical characteristics of the 105 patients, stratified by CKD stage, are described in Table 1. Children in CKD 2–3 were younger and transplant recipients older. There was a higher proportion of glomerular disease in the dialysis group. Calcium, phosphate, and PTH levels, and use of phosphate binder and activated vitamin D were highest in dialysis patients.

### Vitamin A intake

**Fig. 2:**
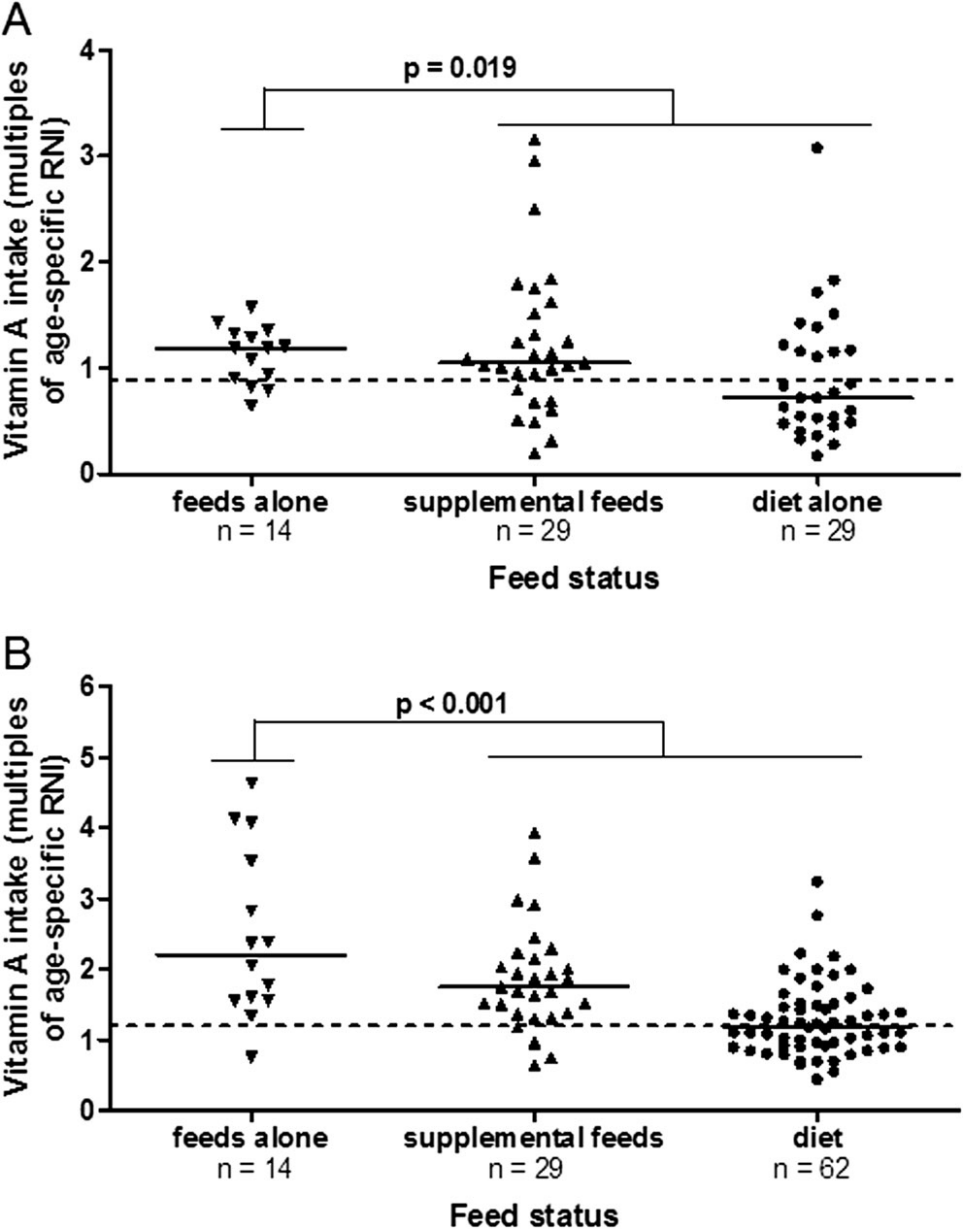
**a** Vitamin A intake is higher in children on supplemental feeds or feeds alone compared with those on a diet. The dietary vitamin A intake is expressed as multiples of the age-specific Reference Nutrient Intake (RNI). **b** Serum retinol levels (expressed as multiples of the age-specific upper limit of normal) were highest in children on supplemental feeds or feeds alone compared with those on a diet alone. The *hyphen* represents the median in each category

Data on vitamin A intake was available in 72 children (69 %): 14 (19 %) were dependent on feeds alone, 29 (40 %) received a combination of diet and supplementary feed, and 29 (40 %) were on diet alone. Children receiving exclusive or supplementary feeds had a higher vitamin A intake compared with those on diet alone (119 % vs. 104 % vs. 71 % of RNI, respectively, *p* = 0.02) (Fig. 2a); 53 % of children exceeded the age-specific RNI for vitamin A intake, with 6 % exceeding twice the RNI; 38 % of children on diet alone exceeded the RNI; 62 % on supplementary feeds and 64 % on feeds alone exceeded the RNI. There was no significant difference in vitamin A intake between CKD groups (*p* = 0.45) (Table 2). Of note, none of the patients received medications containing vitamin A.

### Circulating levels of vitamin A metabolites

In 77 % of children, serum ROH levels were above the upper limit of normal (ULN) for age. A higher dietary vitamin A intake relative to RNI was directly associated with a higher serum ROH level (*p* < 0.001) (Fig. 2b). Higher serum ROH was seen in children on feed alone [3.1 (2.3–5.3) μmol/l], and those on supplementary feed and diet [2.8 (2.3–3.4) μmol/l] compared with those on diet alone [2.5 (1.8–3.3) μmol/l] (*p* < 0.001 between groups). Importantly, dialysis patients were more likely to have higher ROH levels relative to their vitamin A intake: when the vitamin A intake was < 50 % of RNI, 22 % of children on dialysis had increased ROH, whereas only 7 % of children in CKD 2–5 had increased ROH at similar RNI.

**Fig. 3:**
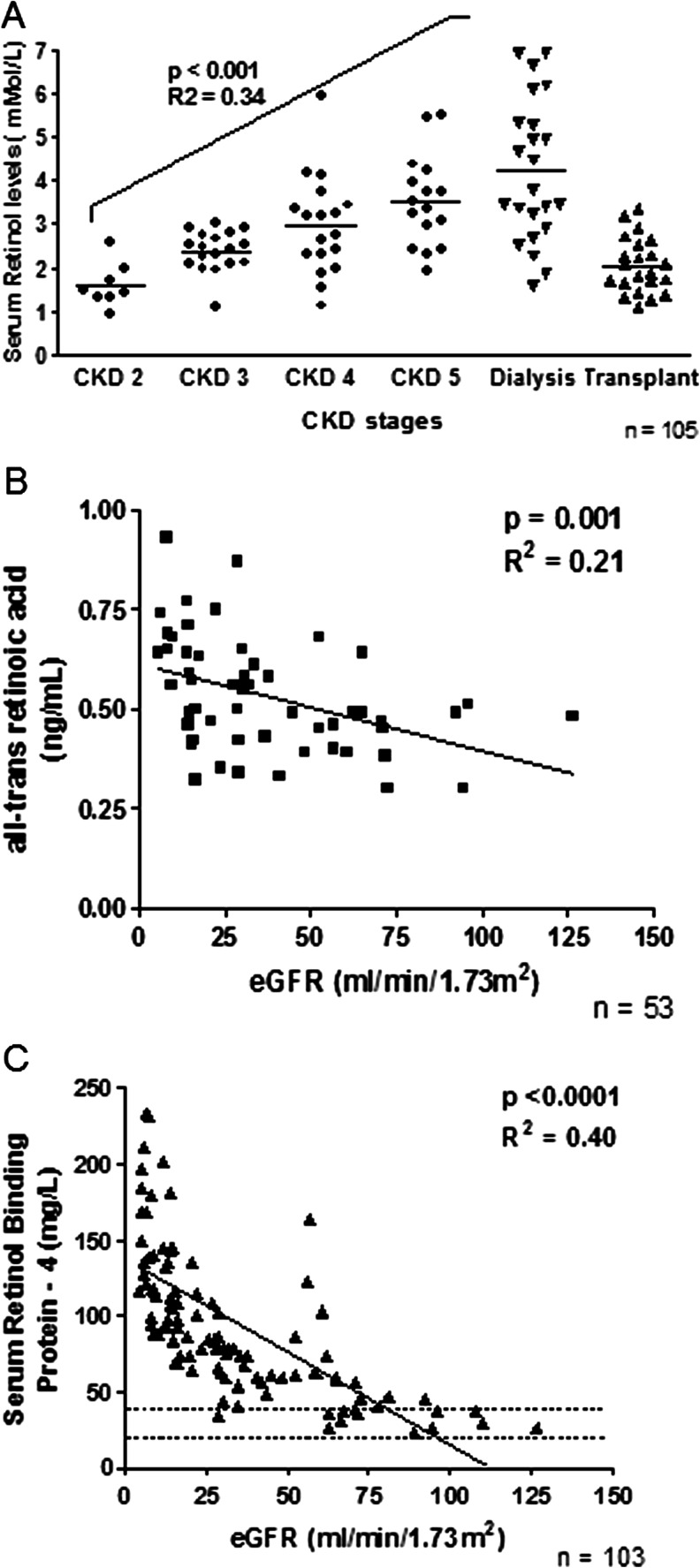
Association of serum retinoid levels with chronic kidney disease (CKD) stages and estimated glomerular filtration rate (eGFR). **a** Serum retinol levels, **b** serum all-trans retinoic acid levels, **c** serum retinol-binding protein-4 levels. *Dotted lines* represent normal range

There was a significant negative association between serum ROH and eGFR in nontransplant patients (*p* < 0.001, *R* ^2^ = 0.34) (Fig. 3a). For every 10 ml/min/1.73 m^2^ fall in eGFR, there was a 13 % rise in serum ROH, even after correcting for vitamin A intake. A similar but weaker association was seen in transplant recipients (*p* = 0.03, *R* ^2^ = 0.2; data not shown).

Levels of at-RA were below the normal range for all patients, with lowest levels in CKD 2–3 and higher levels in dialysis patients. There was an inverse linear association between at-RA and eGFR (*p* = 0.001, *R* ^2^ = 0.21) (Fig. 3b). Among the transplant group, where the median eGFR was 63 ml/min/1.73 m^2^, at-RA levels were comparable with those seen in CKD 2–3 patients [0.46 (0.4–0.58) vs 0.48 (0.4–0.49) ng/ml; *p* = 0.17]. No correlation was observed between eGFR and 13-cis RA (*p* = 0.6). Children in CKD stages 3–5 and on dialysis had high levels of RBP4 (Fig. 3c). Both RBP4 and TTR levels increased with declining GFR (*p* < 0.0001, *R* ^2^ = 0.40; *p* < 0.001, *R* ^2^ = 0.32, respectively) and were highest in dialysis patients. Although there was a linear association between serum ROH and RBP4 (*p* < 0.001, *R* ^2^ = 0.76), molar ratios for ROH:RBP4 showed a significantly different binding pattern at different CKD stages (Table 2), with the lowest ratios in CKD 4–5 and dialysis groups (0.72 and 0.59, respectively) compared with CKD 2–3 patients (0.81; *p* = 0.03);,suggesting relatively higher amounts of free RBP4 (apo-RBP) with advanced CKD. Similarly, an increased RBP4:TTR ratio was seen, suggesting increased amounts of non-TTR-bound RBP4 in dialysis compared with CKD 2–3 patients (1.44 vs. 0.82; *p* = 0.02) (Table 2).

Serum levels of ROH, RBP4, TTR, and RA were not influenced by dialysis modality or duration (data not shown). RBP4 is an adipokine, and elevated RBP4 levels are associated with obesity; however, in our cohort, there was no association between body mass index (BMI), expressed as standard deviation score (SDS) and RBP4 levels or any other serum retinoid levels. RBP4 levels were associated with total cholesterol in transplant recipients (*p* = 0.05, *r* = 0.2). No patient had clinical signs or symptoms of vitamin A toxicity, such as alopecia, pruritus, or raised intracranial pressure.

### Associations with serum albumin-adjusted calcium

**Fig. 4:**
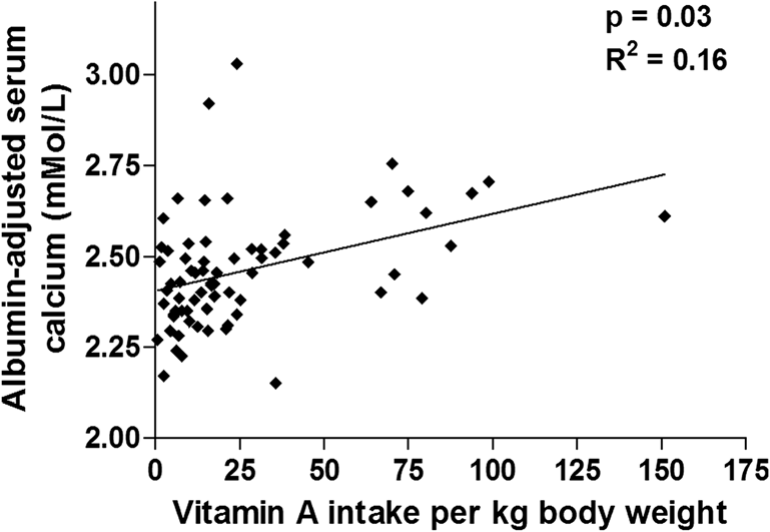
Increased vitamin A intake is associated with serum albumin-adjusted calcium levels

A higher vitamin A intake was associated with higher calcium levels (*p* = 0.03, *R* ^*2*^ = 0.16): for each 10 μg/kg/ per day increase in vitamin A intake, there was a 0.03 mmol/l increase in albumin-adjusted calcium levels (Fig. 4). Notably, there was no association between dietary vitamin A intake and dietary vitamin D or calcium intakes. The total calcium intake (from diet and binders) increased with CKD stage, but only four children (one CKD 5 and three on dialysis) exceeded the RNI for calcium from diet and binders combined (Table 1).

**Fig. 5:**
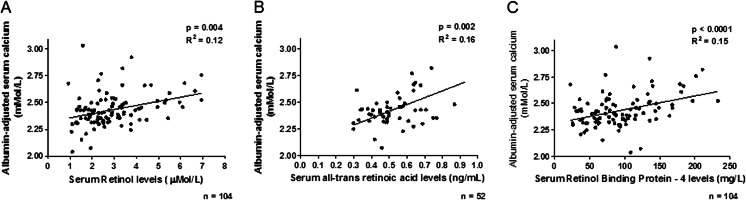
Association of serum retinoid levels with albumin-adjusted calcium levels. **a** Serum retinol levels, **b** serum all-trans retinoic acid levels, **c** serum retinol-binding protein-4 levels

Children with higher ROH levels had significantly higher albumin-adjusted calcium levels (*p* = 0.004; *R* ^2^ = 12) (Fig. 5a). Similarly, there was a positive association between at-RA and albumin-adjusted calcium (*p* = 0.002, *R* ^2^ = 0.16) (Fig. 5b) and RBP4 and albumin-adjusted calcium (*p* < 0.0001, *R* ^2^ = 0.15) (Fig. 5c). No association was seen with 13-cis RA. Dialysis patients with the highest Alkaline phosphatase (ALK) had the lowest RBP4 levels (*p* = 0.04, *r* = 0.22).

Potential predictors of serum albumin-adjusted calcium levels [eGFR, calcium intake (from diet and binders combined), alfacalcidol dose, serum levels of 25-hydroxyvitamin D, vitamin A intake, serum ROH, RBP4, and all-trans RA] were tested. On univariate analysis, there were no significant predictors for calcium levels in the CKD 2–5 or transplant cohorts (and no patient had *p* values < 0.15 for inclusion in a multivariable model). However, among children on dialysis, vitamin A intake (*p* = 0.02, *R* ^2^ = 0.14), alfacalcidol dose (*p* = 0.0003, *R* ^2^ = 0.32), and RBP4 levels (*p* = 0.008, *R* ^2^ = 0.26) were significantly associated with albumin-adjusted calcium. On stepwise linear regression analysis, only alfacalcidol dose and RBP4 levels were significantly associated with albumin-adjusted calcium levels (*p* < 0.004, β = 0.22 and *p* = 0.01, β = 0.11; model *R* ^2^ = 32 %).

## Discussion

In this study, we examined children across a wide range of kidney function and found that hypervitaminosis A is seen even in early CKD and may be attributed to an increased vitamin A intake, particularly through supplementary feeds. High levels of RBP4 were significantly and independently associated with hypercalcemia in dialysis patients. Accumulation of RBP4, as evidenced by an altered molar ratio of ROH:RBP4, may be the stimulus that triggers further hepatic release of ROH [1, 2].

Previous studies in both adults [5–7] and children [8–10] focused on dialysis patients only and show marked increase in serum ROH and RBP4 levels. Pediatric studies show that ROH levels are between two- and fivefold higher than normal in children on dialysis and stress the importance of avoiding vitamin A supplementation in these patients [10, 29]. In the largest study, by Fassinger et al., increased levels of ROH, RBP4, and TTR were reported in all children on dialysis, with results comparable with adult dialysis patients [8]. Conversely, Zwolińska et al. showed low vitamin A levels in children on hemodialysis (HD) [30]. There are no studies reporting on children in predialysis CKD. In this study, we show increased levels of ROH, at-RA, RBP4, and TTR, even in CKD 2 patients, with a strong inverse association with eGFR: for each 10 ml/min/1.73 m^2^ fall in eGFR, there was a 13 % rise in serum ROH. Notably, none of the children in our study received medications containing vitamin A. A previous study implied a similar finding of hypervitaminosis A even with mildly reduced renal function: in kidney donors, even a modest reduction in renal function was associated with increased ROH, RBP4, and TTR levels [31].

Despite a linear increase in ROH and its carrier proteins RBP4 and TTR with declining eGFR, we found altered binding properties of ROH to RBP4 at different CKD stages. The molar ratio of ROH:RBP4 was significantly reduced in CKD 4–5 and dialysis patients compared with CKD 2–3 or transplant recipients, suggesting increased circulating levels of RBP4. In CKD patients, we found that RBP4 levels were substantially increased compared with those of TTR; this is in keeping with previous studies that show increasing serum RBP with declining GFR [32]. Under physiological conditions, the ROH:RBP4 complex has high affinity to TTR, thus protecting RBP from glomerular filtration and renal catabolism [33] (Fig. 1). After releasing ROH into target cells, apo-RBP (RBP without ROH) is filtered through glomeruli and reabsorbed via the megalin–cubilin receptor complex and catabolized [34]. This observation supports an earlier hypothesis that apo-RBP provides a positive feedback signal for hepatic release of the ROH–RBP4 complex [35, 36], leading to a vicious cycle of increased circulating RBP4. In postmenopausal women, increased RBP4 levels have been associated with coronary artery calcification [18]. High ROH and RBP4 serum concentrations have been linked to an increased intima-media thickness, an established surrogate marker for cardiovascular disease [17].

We found that at-RA levels were below the normal range in all CKD patients, suggesting a reduced conversion of ROH to at-RA in CKD, as previously described [1, 2]. Paradoxically, the lowest levels of at-RA were seen in CKD 2–3 and transplant recipients, whereas highest levels were found in dialysis patients. at-RA has not been previously described in CKD patients, and these findings need to be explored in larger patient cohorts. Also, it is not clear why at-RA levels below the normal range are associated with hypercalcemia.

Importantly, vitamin A is not simply one more uremic retention product but has been linked with abnormal osteoclast function and hypercalcemia. Even in individuals with normal renal function, administration of supplements containing vitamin A has been linked with hypercalcemia [37–39]. Receptors for RA are located on both osteoblasts and osteoclasts, indicating that they are direct vitamin A targets [40]. In animal studies, elevated RA can increase serum calcium by suppressing osteoblast activity, stimulating osteoclast formation [22] and differentiation from their postmitotic precursors [23] and reducing osteoid formation [41]. Interestingly, these changes were most marked in the growing skeleton [41]. In a study in postmenopausal women, a weak association between vitamin A intake and fracture risk has been observed [37]. Hypercalcemia can also promote or exacerbate ectopic vascular calcification that is part of CKD–mineral and bone disorder (CKD-MBD) complex. Mice fed a diet high in vitamin A developed aortic valve calcification, with an upregulation of osteogenic genes within the aortic valve leaflets [42]. In previous studies, increased RBP4 levels were associated with coronary artery calcification [20], and increased ROH and RBP4 are linked to an increased intima-media thickness [19]. We found a significant correlation between serum ROH, RBP4, and at-RA levels with albumin-adjusted calcium. Given that there was a very low prevalence of hypercalcemia in our patients, this suggests that vitamin A metabolites may influence serum calcium levels even within the normal range. Also, in patients with unexplained hypercalcemia, particularly those with impaired renal function, vitamin A toxicity should be considered.

We found that dietary vitamin A intake was associated with increased levels of ROH and increased serum calcium levels on univariate analysis, although this lost its significance on multivariate analysis. Previous studies in adults [16, 18] and children [10, 29] show elevated serum ROH in dialysis patients and prompted recommendations to restrict vitamin A supplements. Of note, none of our patients were on vitamin A supplements, but high ROH levels were in seen in 53 % of children and were present from earlier stages of CKD. Notably, 87 % of children in CKD 2–5 and on dialysis had increased ROH levels, even though their vitamin A intake was below the RNI. These data suggest that current recommendations for dietary vitamin A intake in children with CKD, both from KDOQI [12] and UK practice [11], may be too high.

For children with CKD, particularly those < 2 years of age, standard infant or pediatric formulas are often used, either alone or as supplementary feeds. The level of vitamin A fortification in standard feeds, as well as renal-specific feeds, may be too high for children with CKD. Currently available feeds can provide up to twice the RNI for vitamin A, and it is a challenge to reduce intake to the RNI without compromising overall protein and energy intake. Although acceptable vitamin A intake is not known, our data suggest intake below the current RNI may be safe. In two cohorts of healthy breastfed infants [43–45] in whom vitamin A intake was 100 μg/day (29 % of RNI), there were no signs of vitamin A deficiency. Intakes lower than the RNI maintained satisfactory ROH concentrations in healthy preschool children [45].

There are some important limitations of this single-center, cross-sectional study. We were unable to distinguish between sources of dietary vitamin A; retinol absorption from plant and animal sources is highly variable. Given the important associations with RBP4 and hypercalcemia, it would have been useful to measure apo-RBP4 and free ROH. Previous studies show that RBP4 levels are correlated with indices of insulin resistance, which would be interesting to assess in future studies. Finally, we were unable to study the effects of hypercalcemia on cardiovascular structure or function.

In summary, we show that hypervitaminosis A is present from early stages of CKD in children and is associated with increased dietary vitamin A intake, particularly from supplementary feeds. RBP4 levels are significantly and independently associated with hypercalcemia in patients on dialysis. A multicenter study in children across all CKD stages is required in order to determine safe vitamin A intake levels.
